# Antineutrophil cytoplasmic antibodies in infective endocarditis: a case report and systematic review of the literature

**DOI:** 10.1007/s10067-022-06240-w

**Published:** 2022-06-23

**Authors:** Inge C. Van Gool, Jesper Kers, Jaap A. Bakker, Joris I. Rotmans, Y. K. Onno Teng, Martijn P. Bauer

**Affiliations:** 1 Department of Internal Medicine, Leiden University Medical Center, Leiden, The Netherlands; 2 Department of Pathology, Leiden University Medical Center, Leiden, The Netherlands; 3 Department of Pathology, Amsterdam University Medical Center, University of Amsterdam, Amsterdam, The Netherlands; 4 Van ‘t Hoff Institute for Molecular Sciences, University of Amsterdam, Amsterdam, The Netherlands; 5 Department of Clinical Chemistry and Laboratory Medicine, Leiden University Medical Center, Leiden, The Netherlands; 6 Laboratory for Genetic Metabolic Diseases, Amsterdam University Medical Center, Amsterdam, The Netherlands

**Keywords:** ANCA, ANCA-associated vasculitis, Glomerulonephritis, Infective endocarditis, Neutrophil extracellular trap, Pauci-immune glomerulonephritis

## Abstract

Infective endocarditis (IE) may be misdiagnosed as ANCA-associated vasculitis (AAV), especially when antineutrophil cytoplasmic antibodies (ANCA) are detected. Distinguishing IE from AAV is crucial to guide therapy. However, little is known about ANCA positivity in IE patients. We present a case report and systematic review of the literature on patients with ANCA-positive IE, aiming to provide a comprehensive overview of this entity and to aid clinicians in their decisions when encountering a similar case. A systematic review of papers on original cases of ANCA-positive IE without a previous diagnosis of AAV was conducted on PubMed in accordance with PRISMA-IPD guidelines. A predefined set of clinical, laboratory, and kidney biopsy findings was extracted for each patient and presented as a narrative and quantitative synthesis. A total of 74 reports describing 181 patients with ANCA-positive IE were included (a total of 182 cases including our own case). ANCA positivity was found in 18–43% of patients with IE. Patients usually presented with subacute IE (73%) and had positive cytoplasmic ANCA-staining or anti-proteinase-3 antibodies (79%). Kidney function was impaired in 72%; kidney biopsy findings were suggestive of immune complexes in 59%, while showing pauci-immune glomerulonephritis in 37%. All were treated with antibiotics; 39% of patients also received immunosuppressants. During follow-up, 69% of patients became ANCA-negative and no diagnosis of systemic vasculitis was reported. This study reviewed the largest series of patients with ANCA-positive IE thus far and shows the overlap in clinical manifestations between IE and AAV. We therefore emphasize that clinicians should be alert to the possibility of an underlying infection when treating a patient with suspected AAV, even when reassured by ANCA positivity.

**Table:** 

Key Points *• This systematic review describes - to our knowledge - the largest series of patients with ANCA-positive infective endocarditis (IE) thus far (N=182), and shows a high degree of overlap in clinical manifestations between IE and ANCA-associated vasculitis (AAV).* *• ANCA positivity was found in 18-43% of patients with infective endocarditis. Of patients with ANCA-positive IE, the majority (79%) showed cytoplasmic ANCA-staining or anti-PR3-antibodies. We emphasize that clinicians should be alert to the possibility of an underlying infection when treating a patient with suspected AAV, even when reassured by ANCA positivity.* *• In patients with IE and ANCA-associated symptoms such as acute kidney injury, an important clinical challenge is the initiation of immunosuppressive therapy. All patients with data in this series received antibiotics; 39% also received immunosuppressive therapy. In many of these patients, ANCA-associated symptoms resolved or stabilized after infection was treated. ANCA titers became negative in 69% , and a diagnosis of AAV was made in none of the cases. We therefore recommend that (empiric) antibiotic treatment remains the therapeutic cornerstone for ANCA-positive IE patients, while a watchful wait-and-see approach with respect to immunosuppression is advised.*

**Supplementary Information:**

The online version contains supplementary material available at 10.1007/s10067-022-06240-w.

## Introduction

Infective endocarditis (IE) is an uncommon disease with an annual incidence of 3–10 per 100,000 persons. Although relatively rare, it is associated with substantial morbidity and mortality, with mortality in the first year estimated at 25–30% [1]. Early diagnosis and treatment are key to improving clinical outcomes. Diagnosis may, however, be difficult as the clinical manifestations of IE are often non-specific, e.g., (low-grade) fever, malaise, and weight loss. Moreover, presenting symptoms are highly variable, including also vascular and immunological phenomena such as petechiae and glomerulonephritis [2]. In the absence of classic IE manifestations, such findings may point the clinician toward an auto-immune instead of an infectious disease, in particular when antineutrophil cytoplasmic antibodies (ANCAs) directed against proteinase-3 (PR3) or myeloperoxidase (MPO) are detected. These antibodies are important diagnostic markers of ANCA-associated vasculitis (AAV). Distinguishing IE from AAV is crucial to guide appropriate treatment: While immunosuppressive therapy is the gold standard for vasculitis, this may be detrimental in patients with IE, whose treatment should first aim to eradicate the causative pathogen.

Little is known about the incidence of ANCA positivity in patients with IE, their clinical presentation and renal pathology, the use of immunosuppressive therapy, and their prognosis. In the present review, we first present a case of *Streptococcus gallolyticus* endocarditis with ANCAs directed against PR3, who presented with arthritis, purpura, and glomerulonephritis and who was treated with antibiotics and, at a later stage, immunosuppressants. We then describe our systematic review of the literature of cases with ANCA-positive IE, focusing on the clinical presentation, renal pathology, treatment, and outcomes, in order to provide a comprehensive overview and more insight into this disease.

## Case presentation

A 59-year-old woman presented with a 2-month history of fatigue, weight loss, taste change, dyspnea on exertion, a 2–3-week history of spiking fever, and a 1-day history of a painful, swollen left lower leg. Her medical history included an appendectomy almost 50 years ago and an ovariectomy due to benign adhesions, but it was otherwise unremarkable. She did not use any medication. She quit smoking over twenty years ago and denied intravenous drug use. Her dental health was moderate to poor with tooth loss and occasional transient dental pain.

Significant physical findings included a new grade 3/6 systolic heart murmur heard best over the right second intercostal space, edema of the left foot and ankle with impaired dorsiflexion of the ankle joint, a confluent petechial rash of the anterior left lower leg (Online resource 1), and few petechial hemorrhages of the toes.

Laboratory studies revealed normocytic anemia (hemoglobulin 4.5 mmol/l), mean corpuscular volume of 84 fL without evidence of iron-, folic acid-, or vitamin B12 deficiencies, a white blood cell count of 10.33 × 10^9^/L, C-reactive protein level (CRP) of 54 mg/L, erythrocyte sedimentation rate of (ESR) 91 mm/hour, a serum creatinine level of 138 μmol/L, and blood urea nitrogen of 5.4 mmol/L. Urinalysis revealed 248 erythrocytes/μL with dysmorphic red blood cells and red blood cell casts on microscopy. The specific antibody assay was positive for anti-PR3 antibodies (14.7 IU/ml, reference <5.0 IU/ml); anti-MPO antibodies and anti-glomerular basement membrane antibodies were negative. Rheumatoid factor (IgM) was strongly elevated (>200 IU/ml). Tests for anti-nuclear antibodies and antibodies to extractable nuclear antigens were negative. Serum complement C3 and C4 levels were normal. Serum immunoglobulin (Ig) IgG and IgM levels were increased (28.1 g/L and 2.87 g/L, respectively), with levels of IgG-kappa and IgG-lambda M-proteins too low to be quantitated and normal IgA levels.

Electrocardiogram revealed sinus tachycardia with frequent premature atrial complexes, an incomplete right bundle branch block, and signs of left ventricular hypertrophy. A diseased, bicuspid aortic valve with mild regurgitation and an attached mass, reminiscent of but not typical for endocarditis, as well as evidence of coarctation of the aorta were seen on transthoracic and transesophageal echocardiograms. Three blood cultures were positive for *Streptococcus gallolyticus,* subspecies *pasteurianus* (formerly *Streptococcus bovis* biotype II). Renal ultrasound ruled out postrenal obstruction, but did show splenomegaly. Ultrasound of the ankle revealed subcutaneous edema, as seen in cellulitis, but no signs of arthritis or synovitis.

**Fig. 1:**
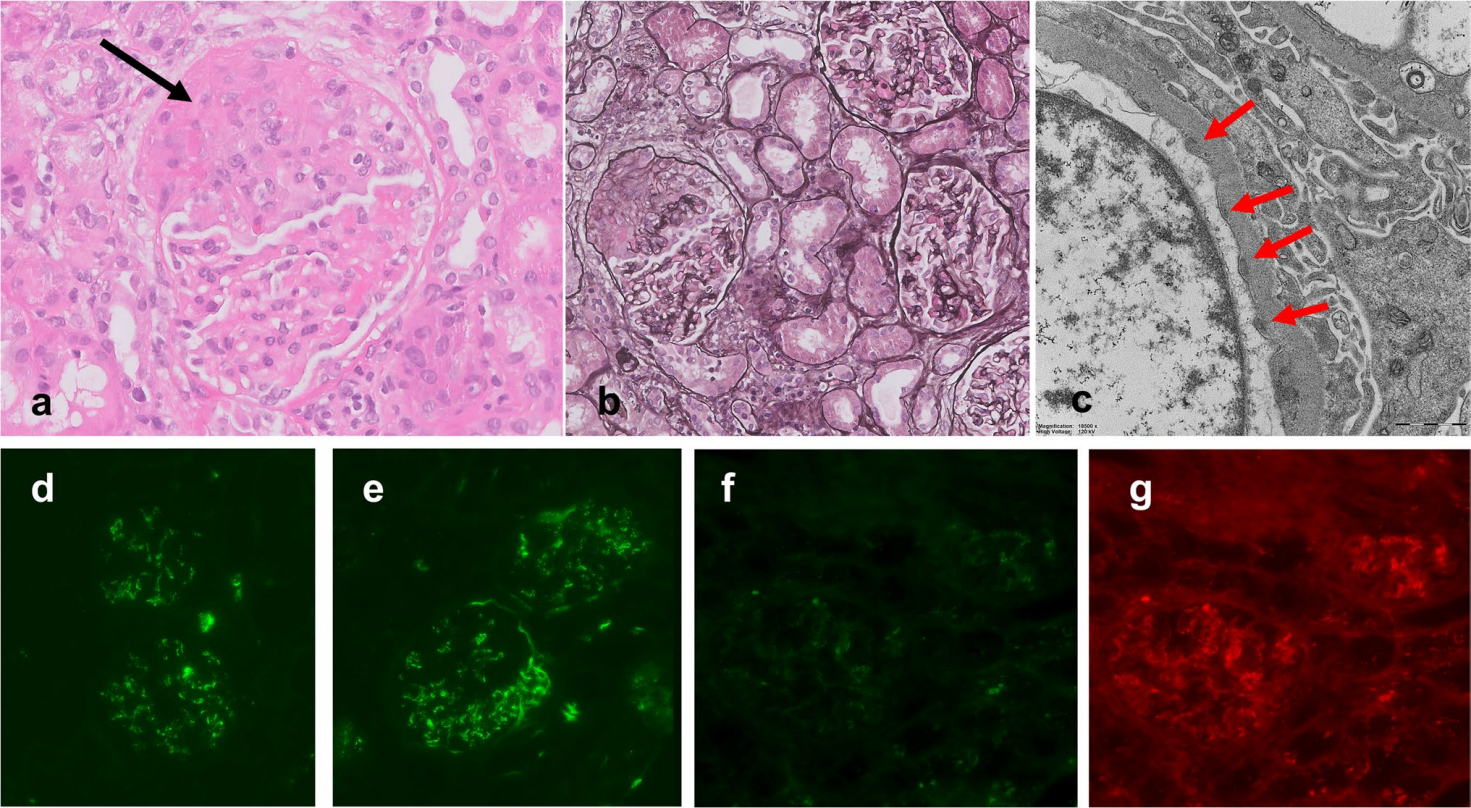
Representative images of renal biopsy findings. Hematoxylin-eosin staining shows a glomerulus with fibrocellular crescent formation (indicated by the black arrow) at 40× magnification (**A**). Jones silver staining shows a fibrocellular crescent without abnormalities in the intact portion of the glomerular basement membrane (**B**, 20× magnification). Electron microscopy showed a few tiny subendothelial depositions (red arrows) without remodeling of the glomerular basement membrane (**C**). Immunofluorescence revealed granular mesangial and focal subendothelial staining of IgM (**D**), C3c (**E**), kappa- (**F**), and lambda light chain (**G**; **D**–**G** all 20× magnification, all scored as maximum intensity 1+).

The patient was diagnosed with anti-PR3 antibody-positive infective endocarditis with a not previously diagnosed congenital heart disease, glomerulonephritis, arthritis, and petechiae. She was treated with intravenous benzylpenicillin for a total of 6 weeks. During the first week of antibiotic treatment, her kidney function remained stable. A kidney biopsy was performed, demonstrating crescentic glomerulonephritis (40% cellular, fibrocellular, and fibrous crescents), focal but active lymphocytic tubulointerstitial inflammation, tubular erythrocyte depositions, and mild interstitial fibrosis/tubular atrophy (Fig. 1A, B). Arteries and arterioles were unremarkable (no endothelialitis/arteriitis). Upon electron microscopy, no subepithelial humps or other glomerular basement membrane abnormalities were observed (Fig. 1C). Immunofluorescence revealed low but notable granular mesangial IgM and C3c staining and kappa- and lambda light chain positivity (all intensity 1+, Fig. 1D–G). IgG, IgA, C1q, and C4d expression was absent. As these findings were not typical of a pauci-immune pattern, both an ANCA-associated glomerulonephritis and an infection-related immune-complex glomerulonephritis were considered.

Oral prednisone of 60 mg/day was started on the eleventh day of antibiotic treatment (by then, six blood cultures had returned negative). The fever, malaise, and swelling and pain of the ankle joint gradually resolved. The patient was discharged after 15 days with continued antibiotic treatment at home (total duration of 6 weeks). Oral prednisone was gradually tapered off and stopped over the course of approximately four months. Kidney function improved slightly with a serum creatinine level of 100 μmol/L eleven months after presentation. Dental consultation revealed periodontitis; the affected molars were removed. The patient was referred to a cardiologist for further analysis of her congenital heart disease. Considering the association of *Streptococcus gallolyticus* bacteremia and colorectal tumors, a colonoscopy was performed, revealing a cT1N0M0 carcinoma of the ascending colon [3]. Subsequently, she underwent a laparoscopic right-sided hemicolectomy four months after presentation. Anti-PR3 antibody titers decreased before and after surgery, but remained elevated at 12.3 IU/ml.

## Systematic review of literature

### Materials and methods

A systematic review of original full-text articles was conducted in accordance with the PRISMA-IPD guidelines [4]. All papers reporting on original cases of ANCA-positive, infective endocarditis without a previous diagnosis of AAV were considered, without search restrictions regarding study design, number of cases described, or publication date. The following predefined dataset for each patient was extracted: age, gender, medical history, symptoms at presentation, time from onset symptoms until diagnosis, findings during a physical examination at admission, results of laboratory tests (echocardiograms), renal biopsies (histological and immunofluorescent), treatment, and clinical outcomes. Data are presented as a narrative and quantitative synthesis with descriptive statistics. Further details regarding the search strategy and data collection can be found in Online resource 2.

### Results

#### Literature search and epidemiology

The literature search is depicted in Online resource 3. A total of 74 reports were included in the systematic review, describing 181 patients with ANCA-positive IE (total study set 182 patients including our case). These included 59 case reports [5–63], 7 case series [64–70], 7 retrospective [71–77], and 1 prospective [78] cohort studies. It is of interest to note that in six of these studies, ANCA testing was performed in cohorts of patients with infective endocarditis, yielding ANCA positivity in 18–43% of cases (median 26%; only the ANCA-positive IE patients of these studies were included in the remainder of the review) [71–75, 78].

Among the 182 cases with ANCA-positive IE, 79% was cytoplasmic ANCA/PR3-positive, compared to 11% perinuclear ANCA/MPO-positive and 8% double-positive. Detailed results of ANCA testing are shown in Online resource 4. Anti-PR3- and/or anti-MPO antibody titers were described in 54 cases: Median increase was 4.5 times the upper reference limit with 50% of titers falling between 2.6 and 8.5 times the upper reference limit, and 40% of cases having titers below 4 times the upper reference limit [6, 7, 9, 10, 13, 16, 24–26, 28, 30–34, 36, 38, 39, 41, 42, 46, 47, 49, 51, 52, 54, 56, 60, 65, 66, 68–70, 74].

#### Clinical characteristics and laboratory findings

Clinical characteristics and laboratory findings of the included cases are summarized in Table 1 [5–78]. Median age was 55 years, ranging from 6 to 83 years (based on 95 cases). The majority of cases were males (79%) and concerned subacute IE (73%, 56/77 cases; ‘subacute’ defined as such by the authors or when symptoms started ≥1 month prior to presentation). Patients often presented with constitutional symptoms (89%) and a new heart murmur (64%). At presentation, the most common laboratory findings were increased inflammatory markers ESR and/or CRP (97%), anemia (89%), and impaired kidney function (72%). One or more auto-antibodies other than ANCA were frequently found (78%, 71/91 patients tested), with the rheumatoid factor being most commonly identified (63%). Seventeen patients were positive for >1 auto-antibody (33%, 19/57 patients). Circulating immune complexes were described in only four patients, with three yielding positive results (two at admission, one during hospital stay). Cardiac ultrasound revealed vegetations in 79%.

**Table 1:** Patient characteristics

	*n/n with data*	*%*
*Clinical findings*		
Male gender	106/133	79
Constitutional symptoms	131/147	89
New heart murmur	61/96	64
Cardiac valve involved		
Aortic valve	42/116	36
Mitral valve	24/116	21
Multiple valves^a^	22/116	19
Other valve(s)^b^	22/116	19
No valve involvement identified	6/116	5
Vegetations on echocardiography^c^	86/109	79
Splenomegaly	34/78	44
Cutaneous manifestations^d^	38/100	38
Vascular phenomena^e^	35/173	20
*Laboratory findings*		
Elevated CRP and/or ESR	91/94	97
Leukocytosis	27/75	36
Anemia	66/74	89
Thrombocytopenia^f^	29/49	59
Impaired kidney function	86/120	72
Urinalysis: microscopic hematuria^g^	80/98	82
Hypocomplementemia	44/65	68
Hypergammaglobulinemia	35/39	90
Cryoglobulins	15/39	38
Auto-antibodies (other than ANCA):		
Rheumatoid factor	55/87	63
Anti-phospholipid antibodies^h^	23/61	38
Anti-nuclear antibodies^i^	24/101	24
*Risk factors for IE*		
Valvular disease	18/60	30
Valve replacement	9/60	15
Congenital heart disease	11/60	18
Poor dentition or recent dental procedure	11/60	18
Intravenous drug use	8/60	13
ICD or intravenous catheter	4/60	8
History of IE	3/60	5

^a^Aortic- and mitral valves were involved in 17 cases (17/116, 14%), mitral- and tricuspid valves in 2 cases (2/116, 2%), mitral-, tricuspid-, and pulmonary valves in 1 case (1/116, 1%), mitral- and aortic valves with abscess of the interventricular septum in 1 case (1/116, 1%), and involvement of tricuspid valve and implantable cardioverter defibrillator (ICD) lead in 1 case (1/116, 1%)

^b^Tricuspid valve was involved in 10 cases (10/116, 9%), pulmonary valve in 2 cases (2/116, 2%). Right-sided IE was reported but not specified in 4 cases (4/116, 3%). A pre-existing ventricular septal defect was affected in 2 cases (2/116, 2%), the right atrium in 3 cases (3/116, 2%), and an ICD lead in 1 case (1/116, 1%)

^c^Transthoracic (82/116, 71%), transesophageal (12/116, 10%%), or both transthoracic and transesophageal echocardiograms (22/116, 19%) were performed. For 9 cases, it was unclear whether vegetations were present or not. Of the 23 cases without vegetations on initial investigations, repeat ultrasounds at a later stage showed vegetations in 43% (*n* = 10)

^d^Cutaneous manifestations included 28 cases with purpuric rash, 4 with petechial rash, 4 with a rash not otherwise specified, 1 with splinter hemorrhages, and 1 with Osler nodes

^e^Vascular phenomena included cerebral infarction (*n* = 6), septic embolism (*n* = 1) or (micro)hemorrhages (*n* = 2), symmetrically decreased perfusion of the frontal lobes (*n* = 2), cerebral vasculitis changes not otherwise specified (*n* = 1), septic pulmonary embolism (*n* = 6), multiple pulmonary nodules and hilar lymphadenopathy resembling polyangiitis (*n* = 1), splenic infarction (*n* = 9), or renal infarction (*n* = 1)

^f^Thrombocytopenia was mild in 18 patients (62%, platelet count 100–150 × 10^9^/L) and severe in one case (3%, <50 × 10^9^/L)

^g^Eight of these patients presented with gross hematuria

^h^Anti-phospholipid antibodies concerned anticardiolipin antibodies (*n* = 10), lupus anticoagulant (*n* = 2), and/or anti-beta-2-glycoprotein I antibodies (*n* = 2; anticardiolipin antibodies and lupus anticoagulant, *n* = 2; anticardiolipin- and anti-beta-2-glycoprotein antibodies, *n* = 5; anti-phospholipid antibodies not otherwise specified, *n* = 2)

^i^Anti-nuclear antibodies including one case with anti-double-stranded DNA antibodies

*IE*, infective endocarditis; *ICD*, implantable cardioverter defibrillator; *CRP*, C-reactive protein; *ESR*, erythrocyte sedimentation rate

#### Pathogens

A micro-organism was detected in 131 of 148 cases with data (89%) [5–45, 47–72, 74, 76–78]. Details regarding the micro-organisms found are listed in Table 2. Gram-positive bacteria were identified in 88 cases (69%), compared to 42 cases with gram-negative bacteria (33%). In one case, blood cultures were positive for gram-positive as well as gram-negative bacteria. Among the gram-positive bacteria, *Streptococcus* and *Staphylococcus* species were identified most frequently, i.e., in 37 (43% of gram-positive) and 30 cases (35%), respectively. Among the gram-negative bacteria, *Bartonella* species were found in 40 cases (93% of gram-negative).

**Table 2:** Micro-organisms identified in ANCA-positive IE (*n* = 131)

**Pathogen**	***N*** **(%)**
Gram-positive	84 (64)
*Staphylococcus* species	28 (21)
*Staphylococcus aureus*^*a*^	23 (17)
Coagulase-negative *Staphylococci*	5 (4)
*Streptococcus* species^b^	35 (27)
Bovis group D streptococci	7 (6)
Viridans group streptococci	17 (13)
*Enterococcus* species^a^	11 (8)
Other gram-positive cocci	6 (5)
*Tropheryma whipplei*	1 (1)
Other	3 (2)
Gram-negative	31 (24)
*Bartonella* species	28 (21)
*Aggregatibacter aphrophilus*	2 (2)
*Coxiella burnetii*	1 (1)
*Candida parapsilosis*^a^	1 (1)
Multiple	15 (11)
*Bartonella quintana & Bartonella henselae*	10 (8)

^a^Six cases with chronic hepatitis infections: two cases with *Staphylococcus aureus* and chronic hepatitis C infections [23, 77], two cases with *Enterococcus faecalis* and chronic hepatitis C infections [20, 37], one with *Abiotiophia defectiva* and chronic untreated hepatitis B infections [9], and one with *Candida parapsilosis* and chronic hepatitis C infections [15]

^b^Categorization according to Facklam [101]

#### Renal biopsy findings

In kidney biopsy, the most commonly identified pattern of injury was crescentic glomerulonephritis (51%), followed by proliferative glomerulonephritis (18%) (Table 3**)** [5–8, 10–16, 19–22, 24–32, 34, 36–39, 43, 44, 47, 49, 51–60, 62–64, 69, 70, 72, 75–77]. Positivity of C1q and/or immunoglobulins, suggestive for immune complexes, was seen in 31% (16/49 cases), while a pauci-immune staining pattern with or without a trace of complement and/or immunoglobulins was reported in 37% (18/49 cases; Online resource 5) [5–7, 10–16, 20, 22, 24–32, 34, 36, 38, 39, 44, 49, 51, 54, 56–58, 60, 62, 63, 69, 70, 72, 75–77]. Electron microscopy showed electron-dense deposits in 69% (16/23 cases) [5, 12, 13, 15, 16, 20, 22, 26, 28, 30, 31, 44, 52–58, 60, 62, 69, 77]. Deposits were most commonly identified in subendothelial and mesangial areas (14/16, 88%). Only one case reported a single small subepithelial hump on electron microscopy [57].

**Table 3:** Light microscopy findings in kidney biopsies of patients with ANCA-positive IE (*n* = 71)

*Predominant pattern of injury*	*N (%)*
Crescentic GN^a^	36 (51)
Proliferative GN	13 (18)
Mesangiocapillary GN	3 (4)
Mesangioproliferative GN	3 (4)
“Infection-associated GN” NOS	2 (3)
Glomerular sclerosis	5 (7)
Pattern not specified or not reported	9 (13)
*Other features*	
Acute tubular necrosis	10 (14)
Tubulointerstitial inflammation	23 (32)
Predominantly polymorphonuclear infiltrate	7 (10)
Predominantly plasma cells	0 (0)
Arteriits/arteriolitis	3 (4)
Interstitial fibrosis and tubular atrophy	14 (20)

^a^When looking at the percentage of affected glomeruli, crescents were seen in 3–70% of glomeruli with a median of 27% (*n* = 15), and capillary necrosis in 3–54% with a median of 20% (*n* = 10)

*IE*, infective endocarditis; *GN*, glomerulonephritis; *NOS*, not otherwise specified

#### Treatment

All cases with treatment details provided (*n* = 110) received antibiotics [5–34, 36–72, 76, 77]. Of these cases, 43 patients (39%) also received immunosuppressive treatment. This consisted of only corticosteroids (*n* = 25, 58%), cyclophosphamide and corticosteroids (*n* = 14, 33%), rituximab and corticosteroids (*n* = 1, 2%), plasmapheresis and corticosteroids (*n* = 1, 2%), and immunosuppressant not otherwise specified (*n* = 2, 5%). In 15% (17/110 cases), immunosuppression was started at a later stage as AAV was suspected. In another 15% (16/110 cases) where immunosuppression was the first treatment and IE was diagnosed later, immunosuppressants were (tapered off and) stopped in 90% (9/10 cases; further details regarding treatments, see Online resource 6). Overall, in patients treated with both antibiotics and immunosuppression, maintenance treatments were given in 14% (*n* = 6), compared to 65% (*n* = 23) without (not reported in 9 cases).

Six patients received intravenous immunoglobulins (IVIG) with reasons for this treatment provided in four cases [7, 13, 58, 66, 69, 76]. In one case, IVIG (2mg/kg) was the initial treatment as the diagnosis was uncertain and kidney biopsy was not possible due to the patient’s clinical condition. When blood cultures returned positive, antibiotics were started [13]. In two patients, corticosteroids and IVIG were started when renal function progressively declined in spite of antibiotics [7, 69]. Finally, one patient who was treated for the suspicion of auto-immune vasculitis with plasmapheresis and corticosteroids was suspected of a complicating infection: Plasmapheresis was stopped, and IVIG started. Immunosuppression was stopped altogether after the diagnosis of IE [58].

#### Outcomes

Regarding clinical outcomes (Table 4), 84% of patients were alive at the last follow-up, which was a median of 6 months after discharge (range 4 days to 6 years based on 53 cases). Only one patient returned after four months with infective endocarditis with blood cultures positive for the same micro-organism; it is, however, unclear whether this concerned a relapse of disease or re-infection as no typing was described [7]. When kidney function was impaired at presentation, this was restored after treatment in 45% (30/67 cases), and improved but not to pre-existent or normal creatinine levels in 28% (19/67). When looking at treatments given, renal function recovered in 53% receiving only antibiotics (17/32 cases), compared to 43% of cases (12/28) treated with antibiotics and immunosuppressants. These data should be interpreted with caution as immunosuppressants were mostly administered in the patients with persistent deterioration of their renal function. During follow-up, ANCA titers became negative in 69%. The median duration of follow-up was 4 months based on 41 cases. No diagnosis of vasculitis was described during follow-up (data available in 48 cases).

**Table 4:** Follow-up data (outcomes, kidney function, ANCA titers) of patients with ANCA-positive IE

	All cases n/n with data (%)	Cases treated with antibiotics alone n/n with data (%)	Cases treated with antibiotics and immunosuppressants n/n with data (%)
*Clinical outcome*			
Dead	21/130 (16)	7/47 (15)	6/35 (17)
*Kidney function*			
Restored to baseline	30/97 (31)	17/46 (37)	12/35 (34)
Improved but not restored	19/97 (20)	11/46 (24)	8/35 (23)
No improvement	18/97 (18)	4/46 (9)	8/35 (23)
No decline at presentation	30/97 (31)	14/46 (30)	7/35 (20)
*ANCA titers*			
Positive	17/55 (31)^b^	12/31 (39)	5/23 (22)
Increased or stable^a^	3/55 (5)	1/31 (3)	2/23 (9)
Decreased^a^	11/55 (20)	9/31 (29)	2/23 (9)
Negative	38/55 (69)	19/31 (61)	18/23 (78)

^a^Change in ANCA titer compared to ANCA titer at presentation

^b^ANCA titers were increased in 1, stable in 2, and not reported in 3 cases

*IE*, infective endocarditis

## Discussion

Antineutrophil cytoplasmic antibodies (ANCA) are a hallmark of a subset of small vessel vasculitides, collectively termed ANCA-associated vasculitis (AAV). ANCAs, however, are not limited to these vasculitides. Indeed, ANCAs are also detected in various other diseases, including rheumatoid arthritis, systemic lupus erythematosus, ulcerative colitis, and Crohn’s disease, and infectious diseases including tuberculosis and endocarditis [79]. We described a case with anti-PR3 antibody-positive infective endocarditis. Multiple case reports and series of this entity have been published; to our knowledge, a comprehensive overview of these cases was lacking. We systematically reviewed the published cases, resulting in the largest series of patients with ANCA-positive IE described thus far (*n* = 182), aiming to provide an improved understanding of this disease and to aid clinicians in their decisions when encountering a similar case.

Previous studies reported features predominantly seen in IE as compared to AAV, namely, extracardiac manifestations limited to skin and kidneys, splenomegaly, hypocomplementemia, dual ANCA positivity (specifically high PR3/low MPO), increased circulating immune complexes and/or immune complex deposition in histological specimen, other positive auto-antibodies, cryoglobulins, and positive blood cultures [45, 76]. Indeed, skin manifestations (mostly concerning purpura), impaired kidney function, and splenomegaly were frequently found in this much larger series of IE cases (38%, 72%, and 44%, respectively), while in AAV often also pulmonary and central nervous system findings were seen, and splenomegaly is rare. This study also confirms hypocomplementemia as a feature predominantly seen in IE (68% in this study, compared to 11–23% previously described in AAV) [80, 81]. While dual anti-PR3- and anti-MPO-positivity in this series was rare (8%) and therefore does not seem discriminative between IE and AAV, low ANCA titers may aid in this distinction. Forty percent of ANCA-positive IE in our analysis had titers below four times the upper limit of normal, which was previously described as a reasonable cut-off point to discriminate between AAV and other diagnosis with 84% of AAV having titers above this cut-off [82]. Increased circulating immune complexes could not be substantiated as this was described in only three cases; C1q- and/or immunoglobulin positivity, suggestive of immune complexes, were, however, a common finding in renal biopsies (59%). Furthermore, despite the high occurrence of other auto-antibody positivity (78%), this does not seem indicative of ANCA-positive IE. Frequencies identified in this study are similar to or lower than those previously reported for AAV: Rheumatoid factor, anti-phospholipid, and anti-nuclear antibodies were found in 63%, 38%, and 24% of ANCA-positive IE, respectively, compared to 39–60%, 21–26%, and 50% of AAV [83–87]. Finally, sex may be added as a discriminative factor, as 79% of ANCA-positive IE cases concerned males, which is in line with male-to-female ratios in IE regardless of ANCA positivity (i.e., 3:2 to 9:1), but higher compared to the 1:1 ratio noted in AAV [88–90].

With pending blood culture results and frequent decline of kidney function, a kidney biopsy was performed in 71 cases, aiming to establish the diagnosis and guide treatment. Similar to the clinical characteristics, much overlap exists in renal pathology findings between glomerulonephritis in (subacute) infective endocarditis and ANCA-mediated glomerulonephritis. One or more features favoring the diagnosis of AAV were commonly identified with crescentic glomerulonephritis reported in 51%, and a negative or pauci-immune immunofluorescence pattern in 37%. Interestingly, in none of the kidney biopsies, an immune infiltrate consisting of predominantly plasma cells is described, while AAV often contains many plasma cells whose role remains to be elucidated. Features favoring the diagnosis of glomerulonephritis associated with IE are the presence of proliferative glomerulonephritis (18%) and of immune complexes and/or complement depositions (63%).

This diagnostic uncertainty was also reflected in the treatment decisions made. While all patients, whose treatment details were provided, received antibiotics at some point in the course of the disease, 39% of patients also received immunosuppressive treatment. This therapy was administered more often to patients with an impaired kidney function at presentation (40% compared to 21% of patients with normal renal function) and, as was the case in our patient, to patients with renal biopsy findings reminiscent of ANCA-mediated glomerulonephritis. From our study, we can deduct that in many of the described cases with ANCA-associated symptoms, such as acute kidney injury, these symptoms resolved or stabilized after the infection was successfully treated. Importantly, no diagnosis or long-lasting features of AAV were reported during reported follow-up of IE patients. Moreover, in 89% of ANCA-positive IE patients, ANCA titers were negative or decreased after a median follow-up of four months. Given the counterproductive effects of immunosuppression in resolving IE, we therefore strongly recommend that (empiric) antibiotic treatment remains the therapeutic cornerstone for ANCA-positive IE patients while a watchful wait-and-see approach with respect to immunosuppression is advised.

The pathogenesis of ANCA in IE, as well as in AAV, is not fully understood. The bacteria causing IE may play a role. Infections induce neutrophil activation and degranulation, and a process called NETosis. In NETosis, neutrophil extracellular traps (NETs), i.e., net-like structures composed of DNA, nuclear and granular proteins including MPO and PR3, are released by neutrophils to trap and kill pathogens. NETs thus express potential auto-antigens and have been shown in mice to stimulate ANCA production [91, 92]. This is supported by the presence of other auto-antibodies directed against NET components in the published IE patients with anti-nuclear antibodies in 24% and anti-phospholipid antibodies in 38% (others such as anti-dsDNA-, anti-histone antibodies described too infrequently to draw conclusions). Moreover, a mechanism called molecular mimicry has been postulated, where bacterial antigens may induce the production of antibodies that also have an affinity for self-antigens [93]. *S. aureus* has been implicated as such an exogenous antigen source, as chronic *S. aureus* nasal colonization has been associated with higher relapse risk in granulomatosis with polyangiitis (a subset of AAV) [94–96]. Furthermore, various pathogens including *S. aureus* include DNA sequences homologous to PR3-antisense DNA, possibly leading to the production of antigens mimicking the complementary peptide (cPR3). In mice, immunization with cPR3 leads to the production of anti-cPR3- as well as anti-PR3-antibodies [97]. Taken together, these mechanisms may explain why 18–43% of patients with IE showed ANCA positivity in our study. Regarding the patient described in the case report, it should be noted that she not only had IE but also had an early stage colon carcinoma. Although malignancy-associated AAV has been very rarely described and pathogenic events causing vasculitis in cancer patients remain poorly understood, a role in or contribution to the clinical manifestations cannot be ruled out [98].

Interestingly, ANCA positivity seemed particularly high in patients with *Bartonella* endocarditis, namely 60% in one study, compared to 23% in gram-positive IE in the same study [74]. In other studies with cohorts consisting predominantly of gram-positive IE, percentages of ANCA positivity were similar (18% in Mahr et al. with 70% gram-positive IE [78]; 24% in Langlois et al. with 80% gram-positive IE [72]). One preclinical study showed that *B. quintana* lipopolysaccharide delayed neutrophil apoptosis, leading to increased antigenic exposure and antibody generation [99]. Whether ANCA positivity is indeed more prevalent among *Bartonella* endocarditis and what the underlying mechanism is, remains to be elucidated.

In this study, all published cases of ANCA-positive IE were reviewed, resulting in – to our knowledge – the largest case series described thus far and the first systematic review. Only case reports, case series, and cohort studies were included in this report yielding a high risk of selection and publication bias. Furthermore, most studies were retrospective in nature with the quality of the studies depending on the availability and accuracy of records; this resulted in many missing data. We should therefore be careful with drawing conclusions. Despite these limitations, considering the relative rarity of both IE and AAV and the absence of higher-quality evidence, this study provides a valuable overview of the characteristics, treatments, and outcomes of patients with ANCA-positive IE. Previous studies showed that immunoassays for PR3- and MPO-ANCAs had a high diagnostic performance to discriminate AAV from disease controls [100]. The control group contains patients with infectious diseases not otherwise specified. As a considerable number of patients with IE are ANCA-positive, we recommend the inclusion specifically of a group of patients with IE in future studies investigating the diagnostic performance of ANCA tests. This study emphasizes that clinicians should be alert to the possibility of an underlying infection when treating a patient with suspected AAV, even when reassured by the presence of ANCA, especially when the clinical condition and/or renal function continue to deteriorate despite (high dose) immunosuppressive therapy.

## Supplementary information

ESM 1 (PNG 1755 kb)

High resolution image (TIF 2477 kb)

ESM 2 (DOCX 15 kb)

ESM 3 (PPTX 43 kb)

ESM 4 (DOCX 16 kb)

ESM 5 (DOCX 14 kb)

ESM 6 (PDF 116 kb)

ESM 7 (DOCX 31 kb)
