# Supplementary figures and images for: Antineutrophil cytoplasmic antibodies in infective endocarditis: a case report and systematic review of the literature
Source: Clin Rheumatol. 2022 Jun 23;41(10):2949–60. doi: 10.1007/s10067-022-06240-w (PMC9485185; doi:10.1007/s10067-022-06240-w)

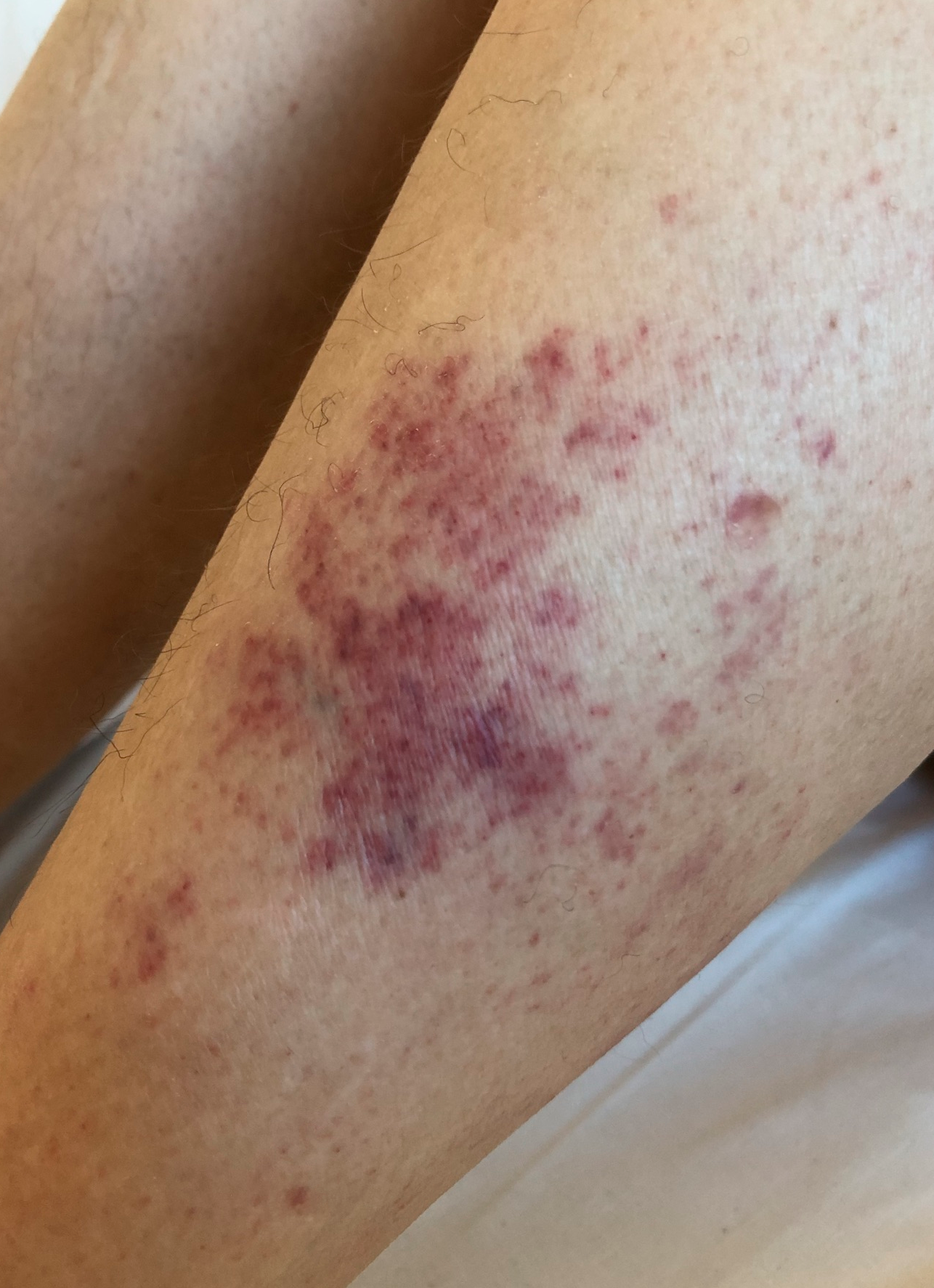

Supplement: Supplementary file 1 — (PNG 1755 kb) [file 10067_2022_6240_Fig2_ESM.png]

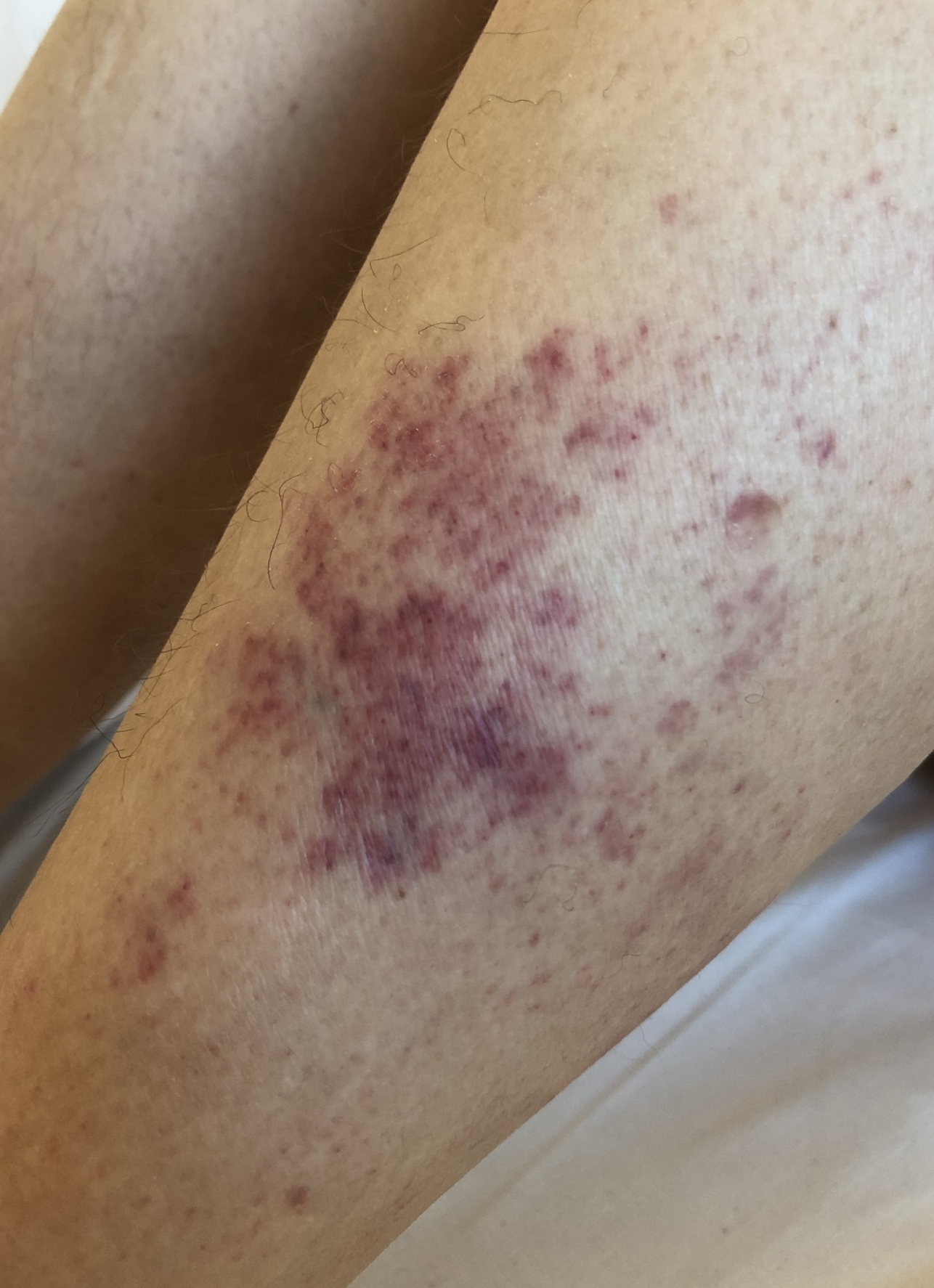

Supplement: Supplementary file 2 — High resolution image (TIF 2477 kb) [file 10067_2022_6240_MOESM1_ESM.tif]
