# Supplementary material for: Antineutrophil cytoplasmic antibodies in infective endocarditis: a case report and systematic review of the literature
Source: Clin Rheumatol. 2022 Jun 23;41(10):2949–60. doi: 10.1007/s10067-022-06240-w (PMC9485185; doi:10.1007/s10067-022-06240-w)
Supplement: Supplementary file 3 — (DOCX 15 kb) [file 10067_2022_6240_MOESM2_ESM.docx]

**Online Resource 2. Supplemental Materials and Methods**

*Search strategy and article selection*

We conducted a systematic search of PubMed on August 8, 2021, using the keywords “endocarditis”, “antineutrophil cytoplasmic antibodies”, “antiproteinase 3”, “MPO” and related entries. This lead to the following search strategy:

("Endocarditis"[Mesh:NoExp] OR "Endocarditis, Bacterial"[Mesh] OR "endocarditis"[tw]) AND ("Antibodies, Antineutrophil Cytoplasmic"[Mesh] OR " Antineutrophil Cytoplasmic Antibod*"[tw] OR "Anti-neutrophil Cytoplasmic Antibod*"[tw] OR "ANCA"[tiab] OR "c-ANCA"[tw] OR "c ANCA"[tw] OR "p-ANCA"[tw] OR "p ANCA"[tw] OR "cANCA "[tw] OR "pANCA"[tw] OR "PR3"[tiab] OR "PR-3"[tw] OR "PR3"[tw] OR "antiproteinase 3"[tiab] OR "antiproteinase-3"[tiab] OR "proteinase 3"[tiab] OR "proteinase-3"[tiab] OR "MPO"[tiab] OR "MPO"[tw]).

We searched the reference lists of articles resulting from the Pubmed search in order to identify additional primary studies. Articles were screened, initially on title and abstract; when suitability for inclusion could not be determined based on title and abstract alone or when no abstract was available, the full-text was assessed. If full-text articles were unavailable, these were requested from the corresponding authors when contact information was available. Only articles in English, Dutch or Romance languages were included. Our case was added to the previously described cases.

*Data collection*

As described in the international consensus on ANCA testing, [1] ANCA was considered positive if immunofluorescence or specific antibody assay was positive, resulting in cytoplasmic ANCA (cANCA)/PR3-positive, perinuclear ANCA (pANCA)/MPO-positive and double-positive cases (PR3- and MPO-positive, cANCA- and pANCA-positive, or cANCA-, pANCA-, PR3-, and MPO-positive). Kidney function was categorized as impaired when it was described as such by the authors or when creatinine levels were above the reported reference value or >100 µmol/L or equivalent. Kidney function was defined as restored when creatinine levels during follow-up normalized or were equal to or lower than the pre-existent levels. Hypocomplementemia was defined as such by the authors or defined by C3 <80mg/dl, C4 <10mg/dl and/or CH50 <30 units/ml. Hypergammaglobulinemia was present when defined as such by the authors or serum IgG was >2000mg/dl, IgA >470mg/dl and/or IgM > 230mg/dl. Streptococcus species were categorized according to Facklam [2].

*References*

1. Bossuyt X, Cohen Tervaert J-W, Arimura Y, Blockmans D, Flores-Suárez LF, Guillevin L, Hellmich B, Jayne D, Jennette JC, Kallenberg CGM, Moiseev S, Novikov P, Radice A, Savige JA, Sinico RA, Specks U, van Paassen P, Zhao M-h, Rasmussen N, Damoiseaux J, Csernok E. Revised 2017 international consensus on testing of ANCAs in granulomatosis with polyangiitis and microscopic polyangiitis. Nature Reviews Rheumatology. 2017;13:683-92.10.1038/nrrheum.2017.140.
2. Facklam R. What happened to the streptococci: overview of taxonomic and nomenclature changes. Clin Microbiol Rev. 2002;15:613-30.10.1128/CMR.15.4.613-630.2002.
