# Supplementary material for: Antineutrophil cytoplasmic antibodies in infective endocarditis: a case report and systematic review of the literature
Source: Clin Rheumatol. 2022 Jun 23;41(10):2949–60. doi: 10.1007/s10067-022-06240-w (PMC9485185; doi:10.1007/s10067-022-06240-w)
Supplement: Supplementary file 4 — (PPTX 43 kb) [file 10067_2022_6240_MOESM3_ESM.pptx]

## Slide 1
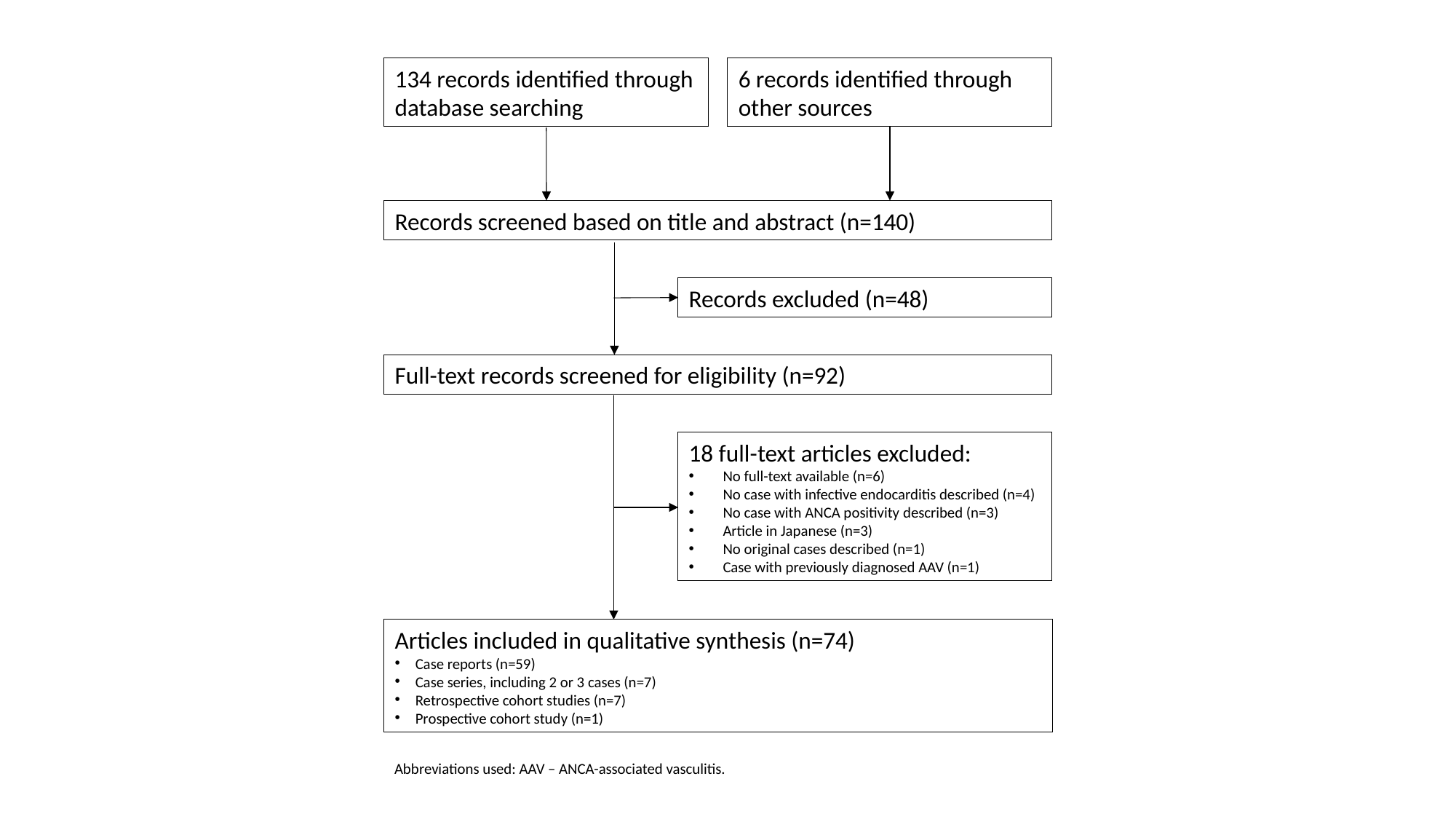

134 records identified through database searching
6 records identified through other sources
Records screened based on title and abstract (n=140)
Records excluded (n=48)
Full-text records screened for eligibility (n=92)
18 full-text articles excluded:
No full-text available (n=6)
No case with infective endocarditis described (n=4)
No case with ANCA positivity described (n=3)
Article in Japanese (n=3)
No original cases described (n=1)
Case with previously diagnosed AAV (n=1)
Articles included in qualitative synthesis (n=74)
Case reports (n=59)
Case series, including 2 or 3 cases (n=7)
Retrospective cohort studies (n=7)
Prospective cohort study (n=1)
Abbreviations used: AAV – ANCA-associated vasculitis.
