# Supplementary material for: Antineutrophil cytoplasmic antibodies in infective endocarditis: a case report and systematic review of the literature
Source: Clin Rheumatol. 2022 Jun 23;41(10):2949–60. doi: 10.1007/s10067-022-06240-w (PMC9485185; doi:10.1007/s10067-022-06240-w)
Supplement: Supplementary file 5 — (DOCX 16 kb) [file 10067_2022_6240_MOESM4_ESM.docx]

**Online Resource 4. Antineutrophil cytoplasmic antibodies (ANCA) in cases with infective endocarditis (N=171)^a^**

| **Immunofluorescence** | | | **Specific antibody test** | | **N (%)** | **References** |
| --- | --- | --- | --- | --- | --- | --- |
| **cANCA** | **pANCA** | **ANCA NOS** | **PR3** | **MPO** |  |  |
| NR | NR | NR | **+** | **-** | 52 (31)^b^ | ^12, 15, 22, 26, 27, 33, 34, 35, 39, 50, 54, 55, 66, 67, 72, 74, our case^ |
| **+** | - | - | **+** | - | 47 (27) | ^8, 9, 13, 14, 16, 19, 23, 24, 29, 31, 37, 38, 40, 42, 43, 48, 52, 56, 57, 60, 69-71, 75-79^ |
| **+** | - | - | NR | NR | 14 (8) | ^28, 44, 46, 51, 65, 79^ |
| **+** | - | - | - | - | 11 (6) | ^36, 75, 76^ |
| - | - | - | **+** | - | 9 (5) | ^6, 7, 10, 17, 47, 61, 65, 73, 79^ |
| - | **+**^c^ | - | NR | NR | 7 (4) | ^20, 62, 79^ |
| **+** | - | - | **+** | **+** | 6 (3) | ^32, 45, 75, 77^ |
| - | **+** | - | - | **+** | 4 (2) | ^76, 77, 79^ |
| - | **+** | - | - | - | 4 (2) | ^41, 64, 76^ |
| NR | NR | NR | **+** | **+** | 4 (2) | ^25. 63, 68^ |
| **+** | - | - | - | **+** | 2 (1) | ^79^ |
| - | - | **+** | **+** | - | 2 (1) | ^11, 59^ |
| - | - | **+**^d^ | NR | NR | 2 (1) | ^18, 49^ |
| - | - | **+** | - | - | 1 (1) | ^76^ |
| **+** | **+** | - | **+** | **+** | 1 (1) | ^53^ |
| NR | - | NR | **+** | - | 1 (1) | ^21^ |
| - | - | - | **+** | **+** | 1 (1) | ^76^ |
| - | - | - | - | **+** | 1 (1) | ^79^ |
| **+** | **+** | - | NR | NR | 1 (1) | ^30^ |
| NR | NR | NR | - | **+** | 1 (1) | ^58^ |

^a^ Eleven cases, described by Langlois et al, [73] are not included in the table as no individual data was available. For these cases, 6 showed cANCA- and 5 pANCA-positivity in immunofluorescence; 3 were PR3-positive, 1 MPO-positive, and 2 PR3- and MPO-positive in specific antibody test.

^b^ Immunofluorescence results of one case [55] were inconclusive.

^c^ Atypical pANCA was found in one case [62].

^d^ ANCA was described as atypical in one case [18].

Abbreviations used: cANCA – cytoplasmic ANCA staining pattern; pANCA – perinuclear ANCA staining pattern; PR3 – anti-proteinase 3 antibody; MPO – anti-myeloperoxidase antibody; NOS – not otherwise specified, NR – not reported.
