# Supplementary material for: Antineutrophil cytoplasmic antibodies in infective endocarditis: a case report and systematic review of the literature
Source: Clin Rheumatol. 2022 Jun 23;41(10):2949–60. doi: 10.1007/s10067-022-06240-w (PMC9485185; doi:10.1007/s10067-022-06240-w)
Supplement: Supplementary file 6 — (DOCX 14 kb) [file 10067_2022_6240_MOESM5_ESM.docx]

**Online Resource 5. Kidney biopsy immunohistochemistry and/or immunofluorescence results (n=49)**

| **Immunoreactant** | **Staining intensity**^a^ | | | | | **Total positive/ tested (%)** |
| --- | --- | --- | --- | --- | --- | --- |
|  | **Negative** | **Positive** | | | |  |
|  | **N (%)** | **1+**  **N (%)** | **2+**  **N (%)** | **3+**  **N (%)** | **NR**  **N (%)** |  |
| C3 | 14 (30) | 4 (9) | 3 (7) | 7 (15) | 18 (39) | 32/46 (70) |
| C1q | 17 (53) | 1 (3) | 5 (16) | 2 (6) | 7 (22) | 15/32 (47) |
| IgG | 18 (56) | 7 (22) | 2 (6) | 1 (3) | 4 (13) | 14/32 (44) |
| IgM | 15 (37) | 4 (10) | 2 (5) | 6 (14) | 14 (34) | 26/41 (63) |
| IgA | 17 (59) | 6 (20) | 2 (7) | 0 (0) | 4 (14) | 12/29 (41) |

^a^ Staining intensity categorized as 1+ (either described as 1+ or as weak, faint or trace), 2+ (described as 2+, moderate or mild), or 3+ (described as 3+, strong, bright, or intense).

Abbreviations used: NR – not reported, IF – immunofluorescence, IHC - immunohistochemistry , Ig – immunoglobulin
