# Supplementary material for: Antineutrophil cytoplasmic antibodies in infective endocarditis: a case report and systematic review of the literature
Source: Clin Rheumatol. 2022 Jun 23;41(10):2949–60. doi: 10.1007/s10067-022-06240-w (PMC9485185; doi:10.1007/s10067-022-06240-w)
Supplement: Supplementary file 7 — (PDF 116 kb) [file 10067_2022_6240_MOESM6_ESM.pdf]

Online Resource 6. Flow diagram of treatments given with details regarding treatment rationale and/or chronology

n=110
Patients with treatment details

n=43 (39%)
Patients receiving antibiotics and immunosuppressants

n=67 (61%)
Patients receiving only antibiotics

n=20 (18%)
Antibiotics first, immunosuppressants later

Reasons to start immunosuppressants:

- n=17 AAV suspected
    - n=9 No clinical improvement or clinical and/or renal deterioration under antibiotics
    - n=6 Renal biopsy results combined with a declining of not improving kidney function
    - n=1 Uncertainty of reliably excluding AAV
    - n=1 Unclear
- n=1 Autoimmune hemolytic anemia
- n=2 Unclear

n=16 (15%)
Immunosuppressants first, antibiotics later

When IE was diagnosed, immunosuppressants were:

- n=6 stopped
- n=3 tapered off and stopped
- n=1 continued
- n=6 unclear

n=7 (6%)
Chronology of treatments unknown
